# A Rare Complication of Lichen Simplex Chronicus Following Deep Inferior Epigastric Perforator Flap Reconstruction

**DOI:** 10.7759/cureus.95331

**Published:** 2025-10-24

**Authors:** Laura M Saunders, Matthew Fung, David Middleton, Clare McGalie, Norah P Scally

**Affiliations:** 1 School of Medicine, Dentistry and Biomedical Sciences, Queen's University Belfast, Belfast, GBR; 2 Department of Dermatology, Craigavon Area Hospital, Portadown, GBR; 3 Department of Histopathology, Craigavon Area Hospital, Portadown, GBR; 4 Department of Breast Surgery, Craigavon Area Hospital, Portadown, GBR

**Keywords:** breast cancer management, diep flap, emollient therapy, lichen simplex chronicus, post surgery complication, topical steroid

## Abstract

Autologous breast reconstruction using deep inferior epigastric perforator (DIEP) flaps is widely regarded as the gold standard technique, offering excellent aesthetic outcomes and reduced donor site morbidity. However, dermatological complications affecting either the reconstructed breast or donor site are infrequently reported and often under-recognized. This manuscript aims to raise awareness of a rare dermatological complication following DIEP flap reconstruction and to discuss its diagnostic and therapeutic considerations. We report a case of a 49-year-old woman who developed a persistent, pruritic rash of the neo breast and abdominal donor site following DIEP flap reconstruction. Surgery formed part of the treatment of a T1aN0M0 invasive ductal carcinoma of the right breast. The patient did not require post-surgical radiotherapy. A biopsy confirmed the diagnosis of lichen simplex chronicus (LSC), a chronic pruritic dermatosis characterized by thickened, scaly plaques resulting from repetitive scratching. To our knowledge, this is the first reported case of LSC occurring specifically within the skin of the transplanted DIEP flap and its donor site. We discuss diagnosis, potential contributing factors - including surgical trauma and possible allergic contact dermatitis - and outline the successful treatment approach using topical corticosteroids and emollients. This case highlights a rare dermatological complication following autologous breast reconstruction and the importance of considering LSC in the differential diagnosis of post-surgical rashes.

## Introduction

Breast reconstruction remains a vital component of care for women undergoing mastectomy, whether for prophylactic reasons or as part of breast cancer treatment. Among autologous reconstruction techniques, the deep inferior epigastric perforator (DIEP) flap has become the most commonly performed procedure, particularly for immediate reconstruction [1]. Compared with the traditional pedicled transverse rectus abdominis myocutaneous (pTRAM) flap, the DIEP flap offers several advantages, including reduced donor-site morbidity, most notably a lower incidence of abdominal wall hernia (1% vs 16%) and fat necrosis (17.7% vs 58.5%), as well as preservation of abdominal muscle integrity [2]. DIEP flap reconstruction is also associated with a shorter postoperative inpatient stay. However, due to the complexity of the microsurgical dissection involved, DIEP procedures typically require a longer operative time compared with pTRAM surgery (five hours 53 minutes vs four hours 46 minutes, respectively) [2].

Despite these benefits, DIEP flap reconstruction is associated with complications such as partial or total flap loss, vascular thrombosis, fat necrosis, and donor-site seroma or haematoma [3]. Overall, the DIEP flap offers superior aesthetic and functional outcomes, with reduced long-term donor-site complications and higher postoperative patient satisfaction [4], albeit at the cost of a more complex and time-consuming procedure.

## Case presentation

A 49-year-old woman underwent a completion mastectomy and immediate DIEP flap reconstruction in December 2023 for a T1aN0M0 invasive ductal carcinoma with extensive ductal carcinoma in situ (DCIS) of the infero-medial right breast. Her early postoperative course was notable for a small area of superficial dehiscence at the abdominal donor site three weeks after surgery, which was treated with a PICO negative pressure dressing and healed fully by January 2024 without signs of deep collection or active infection.

**Figure 1:**
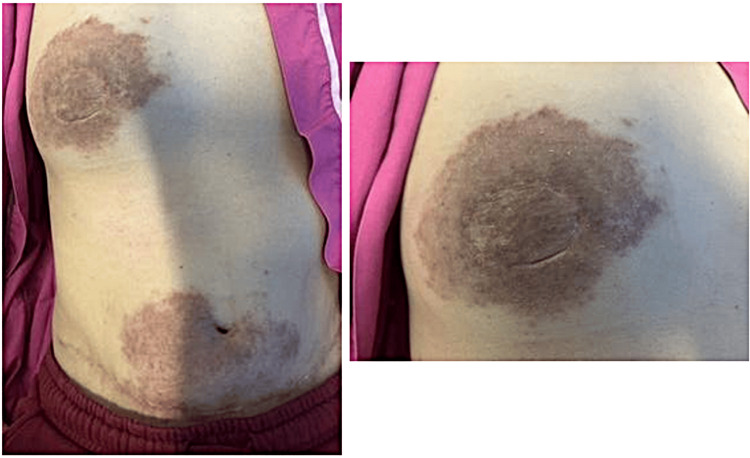
(Left) Hyperpigmented rash of the right breast and periumbilical region around the surgical scar post-DIEP flap reconstruction. (Right) Close-up of the rash on the right breast. DIEP - Deep Inferior Epigastric Perforator

In January 2024, she began applying Bio-Oil once daily to the abdominal scar. After a symptom-free interval of approximately three months, she presented in April 2024 with a two-week history of an intensely pruritic, mildly painful rash. The lesions were distributed along both the abdominal incision and the neobreast crease, evolving from faint hyperpigmented macules to smooth, plaque-like areas with peripheral spread but no overt vesiculation or crusting (Figure 1). The plaques were warm to the touch but non-tender, and there was no regional lymphadenopathy in either axilla.

Her past medical history was unremarkable; she was commenced on oral tamoxifen 20 mg once daily in June 2023 and will continue this for five years. She has no known drug or contact allergies, is a non-smoker, does not consume alcohol, and works indoors in a supermarket with minimal ultraviolet exposure. There is no family history of breast cancer or dermatological disease.

Examination

On examination, the flap remained well perfused throughout her journey, with normal capillary refill. Over the lower abdominal wall, adjacent to the DIEP scar and encircling the umbilicus, as well as on the neobreast, there was a brawny, erythematous, and dry rash. Although an anterior abdominal mesh was in situ, the rash was not confined to the mesh area. The lesions were sharply demarcated, hyperpigmented, lichenified plaques extending 5-6 cm beyond the incision lines, smooth on palpation, and lacking secondary changes such as vesicles, crusts, or excoriations.

Investigations

**Figure 2:**
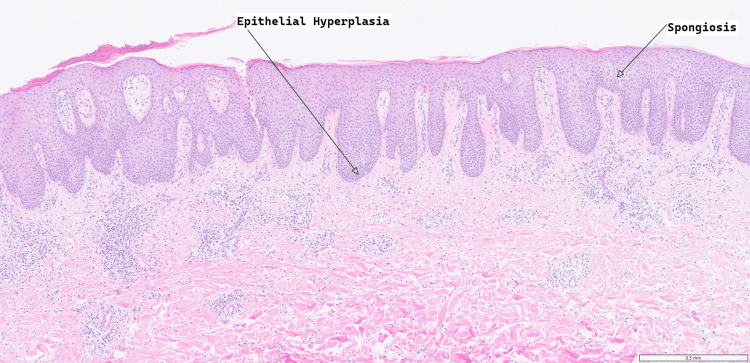
Haematoxylin and eosin-stained section at a x5 magnification, showing epidermal hyperplasia and spongiosis. These histological features are consistent with lichen simplex chronicus.

**Figure 3:**
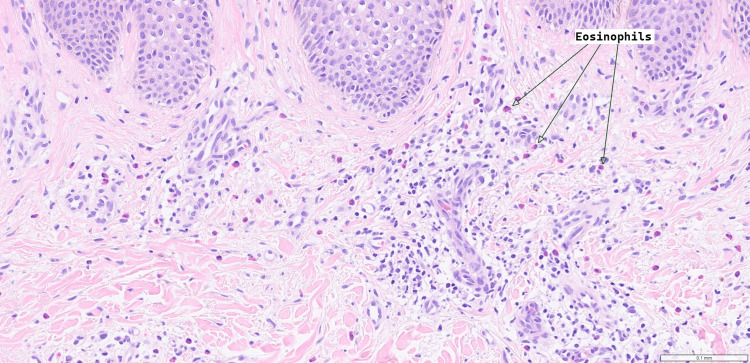
A mixed inflammatory infiltrate is seen in the dermis, including eosinophils with image magnification x20.

Differential diagnoses for this rash included allergic contact dermatitis, chronic eczema, and post-surgical neuropathic pruritus. The first biopsy, taken from the abdominal donor site, was non-diagnostic, with the infiltrate containing lymphocytes, histiocytes, and some eosinophils. There was a mild-to-moderate inflammatory cell infiltrate centred on the blood vessels. Skin scraping identified no fungal, parasitic organisms, or malignancy. The second biopsy, also taken from the abdominal donor site one month after the appearance of the rash, revealed variable parakeratosis and fibrinoinflammatory crust overlying epidermis in which there is psoriasiform hyperplasia. There was spongiosis and a scattered eosinophilic infiltrate within the epidermis. Within the dermis, there was a more florid chronic inflammatory cell infiltrate in a perivascular distribution and prominent eosinophils (Figures 2-3). This was diagnostic of lichen simplex chronicus, thought to be allergic or drug-mediated due to the prominence of eosinophilic infiltration. Blood tests (thyroid-stimulating hormone (TSH), thyroxine (T4), urea and electrolytes (U&E), full blood count (FBC), liver function tests (LFTs)) were also performed to exclude systemic causes of the patient's symptoms.

Treatment

**Figure 4:**
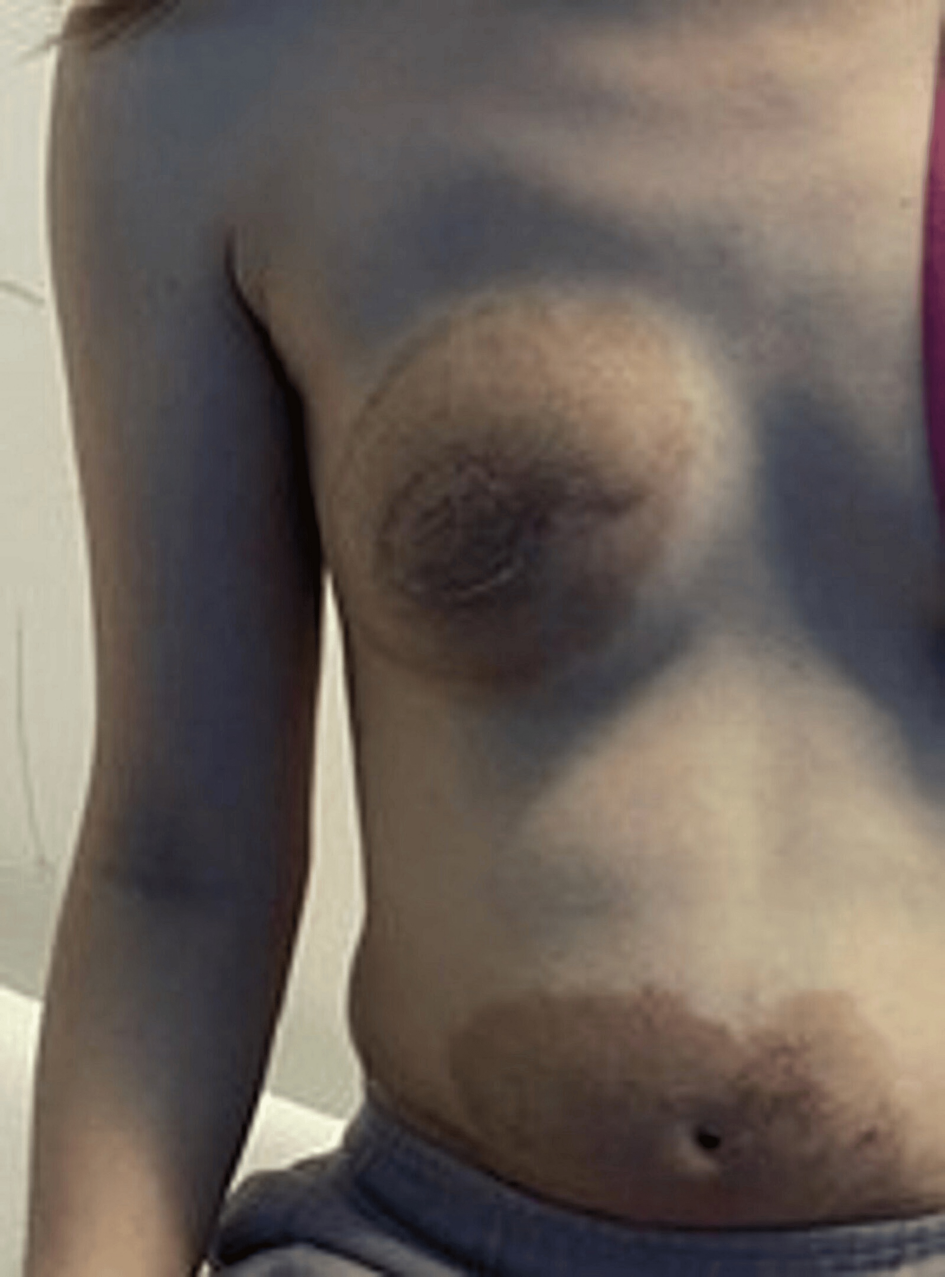
A follow-up picture taken six months after starting treatment. The rash is less erythematous, paler around the edges, and more brawny in colour.

The patient was instructed to stop using Bio-Oil. A trial of 50 micrograms of calcipotriol with 0.5 mg of betamethasone diapropionate foam once daily for two weeks successfully reduced the hyperpigmentation and pruritus; however, upon stopping the application, the rash and symptoms reappeared (Figure 4). A further month’s use of Enstilar foam, along with liberal use of emollient creams (Dermol), has successfully treated the rash, and the patient has since been symptom-free.

Outcome and follow-up

The patient is currently well and fully asymptomatic, able to return to work and hobbies with a full quality of life compared to prior surgical intervention and LSC. Secondary prevention for LSC by applying liberal emollient use to help maintain an intact epidermal layer has been commenced with good compliance from the patient.

## Discussion

LSC is a chronic pruritic dermatosis characterised by well-demarcated, lichenified, scaly patches of skin associated with itch [5]. LSC is thought to be due to the stimulation of nerve endings in the epidermis due to disruption of the epidermal barrier or the environment (e.g., surgical incision), leading to the sensation of itch, which propagates a chronic itch-scratch cycle [6]. Scratching increases lichenification, stimulating nerve endings and resulting in further increased itch and subsequent scratching. LSC can occur as a primary idiopathic condition or secondary process in response to underlying pruritic dermatosis [7]. Although LSC is commonly seen in dermatological practice, its occurrence in the context of autologous flap reconstruction, particularly following DIEP flap surgery, has not been widely reported in the literature.

This case is significant as, to our knowledge, it represents the first documented occurrence of LSC developing in both the neobreast and abdominal donor site following DIEP reconstruction after mastectomy. This highlights a rare but important post-surgical dermatologic complication in flap-based breast reconstruction.

The development of LSC in this case may be multifactorial. Surgical trauma alone can disrupt the skin barrier and local cutaneous innervation, predisposing to chronic pruritus [8]. Furthermore, the process of flap elevation and transplantation may alter local neurovascular structures, contributing to the development of dysaesthesia [9].

A potential contributing factor to the development of LSC is allergic contact dermatitis (ACD) to materials encountered peri- and post-operatively. For example, surgical dressings, suture materials, or topical preparations (silicone-based scar treatments or bio-oils) could stimulate an inflammatory response [10]. Persistent inflammation may have resulted in secondary LSC due to repetitive scratching and compromise of the skin barrier [11]. While ACD and LSC can be difficult to distinguish clinically, a skin biopsy in this case supported a diagnosis of LSC [12].

Although LSC was confirmed histologically, the differential diagnosis included ACD, chronic eczema, and post-surgical neuropathic pruritus. Given the temporal association with surgery and the use of topical agents such as Bio-Oil, ACD could have served as a primary inciting factor leading to secondary LSC [13]. Patch testing could be considered in future similar presentations to explore this possibility [14].

There is limited precedent for LSC occurring in the context of skin grafts or flap reconstruction. Xu et al. reported a case of LSC developing 17 years after retroauricular skin transplantation following a scald injury, suggesting that transplanted or injured skin may be uniquely susceptible to chronic pruritic conditions [15]. While their case presentation differs in context and anatomical location, it underscores the susceptibility of grafted skin to neurosensory changes and chronic pruritic sequelae.

LSC can have a significant impact on quality of life due to persistent itch, discomfort, and the visibility of the affected skin, particularly in cosmetically or psychologically sensitive areas such as the breast [16]. Patients may experience embarrassment, sleep disturbances, and impaired psychosocial functioning [17]. In post-mastectomy patients, this can be particularly distressing, compounding the emotional burden of a cancer diagnosis, treatment, and alteration in body image.

Treatment of LSC focuses on attempting to break the itch-scratch cycle and reduce local inflammation. First-line treatment includes potent topical corticosteroids often applied under occlusion [18]. In this case, the patient was successfully treated with a potent steroid foam combined with a regular emollient. Other treatment options include skin coverage, bandage occlusion, steroid injections, topical calcineurin inhibitors, and tar preparations can all help reduce inflammation [19]. Cooling creams and Capsaicin cream can help manage the itch, which is beneficial to prevent neuropathic itch [20].

## Conclusions

This case highlights a rare dermatological incidence of LSC confined to the abdominal scar and neobreast following DIEP flap reconstruction, emphasising the need for awareness of this potential postoperative complication. Given the physical and psychological impact on patient comfort and quality of life, early recognition and management are paramount. Further research into the cutaneous complications of autologous flap surgery may further our understanding of such complications and inform preventative strategies.
